# Decolorization of mordant yellow 1 using *Aspergillus sp*. TS-A CGMCC 12964 by biosorption and biodegradation

**DOI:** 10.1080/21655979.2018.1472465

**Published:** 2018-07-11

**Authors:** Yanshun Kang, Xiaolin Xu, Huiran Pan, Jing Tian, Weihua Tang, Siqi Liu

**Affiliations:** Key Laboratory for Green Processing of Chemical Engineering of Xinjiang Bingtuan/School of Chemistry and Chemical Engineering, Shihezi University, Shihezi, PR China

**Keywords:** *Aspergillus*, Azo dye, biosorption, biodegradation, mycelia, enzymes

## Abstract

In this report, the decolorization features of extracellular enzymes and mycelia separately prepared from *Aspergillus sp*. TS-A CGMCC 12,964 (120 h) were investigated. The fermentation broth of TS-A degraded 98.6% of Mordant Yellow 1 (50 mg/L) at an initial pH 6 within 1 h with over 70% of the dye (50 mg/L) degraded by extracellular enzymes and 18.8% removed by live mycelia. The degradation products of the dye were analyzed by UV-Vis and FTIR spectra. The decolorization rates of extracellular enzymes and mycelia were examined under different contact periods, dye concentrations and pH values. The extracellular enzymes exhibited excellent degradation activity under weak acidic conditions. In addition, biosorption models of mycelia fitted well the Langmuir isotherm model and the pseudo-second-order kinetic equation. Although the decolorization process was achieved through the synergistic effects of mycelia and extracellular enzymes, decolorization was dominated by the biodegradation activity of the extracellular enzymes from TS-A.

## Introduction

Azo dyes represent the majority of synthetic dyes in the textile industry and are used widely because of their wide spectrum of colors, facile attachment to fibers and low cost [1,2]. However, the discharge of dye wastewater into the environment is undesirable because the dyes possess intensive colors and are a serious health hazard [2,3]. Synthetic dyes and their breakdown products present in textile industry wastewaters have become a major pollution source with about 280,000 tons of textile dyes discharged in such industrial effluents every year [4]. Therefore, there is an urgent need to remove dyes from the effluents before discharging the wastewater into the natural environment.

Compared with physical and chemical methods, biological methods are widely used to remediate dyes present in effluents because of their high efficiency, low cost and secondary pollution [2]. Decolorization by microorganisms has focused on using bacteria, fungi, algae and yeasts, which absorb and/or degrade a wide range of azo dyes [5–7]. Traditional technologies have used bacteria to remove dyes because of their strong adaptability and high activity. However, azo dyes are generally resistant to degradation under aerobic conditions using bacteria; thus, degradation by bacteria requires alternating between anaerobic and aerobic conditions [2,8,9]. Moreover, light intensity and the concentration of CO_2_ are limiting factors for azo dye decolorization by algae [7]. Substantial information has accumulated over the last few years that describes the activity of ligninolytic fungi, especially white rot fungi, which has been shown to degrade multiple types of synthetic dyes [4,6]. Interestingly, fungi possess ligninolytic enzymes that degrade complex dyes, including laccase (Lac), manganese peroxidase (MnP) and lignin peroxidase (LiP) [10]. Among these ligninolytic enzymes, Lac is a copper-containing enzyme that oxidizes various benzene and non-benzene compounds using molecular oxygen as the electron acceptor [10,11]. For example, Lac produced by *Trichoderma lixii* F21 is involved in the conversion of polar dye ARS and no-polar dye QGSS to nontoxic intermediate micromolecules [12]. MnP oxidizes Mn^2+^ into Mn^3+^ in the presence of H_2_O_2_ and the stable Mn^3+^ formed functions as an oxidizer of pollutants [13]. MnP immobilized by chitosan achieved a 97.31% degradation rate of dye effluents, and reduced Chemical Oxygen Demand (COD), Total Organic Carbon (TOC) and Biochemical Oxygen Demand (BOD) at degradation values of 82.40, 78.30 and 91.7%, respectively [14]. LiP degraded most recalcitrant aromatic compounds when veratryl alcohol (VA) acted as a redox mediator [15]. The white rot fungus *Phanerochaete chrysosporium* showed complete decolorization of Direct Red-80 because of its highly active LiP [16]. Apart from white-rot fungi, other fungi such as *Aspergillus niger, Scheffersomyces spartinae* and *Galactomyces geotrichum* also degrade and/or adsorb a diverse range of dyes [17–21]. Methyl orange was degraded by the blue laccase from *Aspergillus ochraceus* NCIM-1146 and its intermediates such as p-N,N’-dimethylamine phenyldiazine and p-hydroxybenzene sulfonic acid were confirmed by FTIR (Fourier transform infrared spectroscopy), HPLC and GC-MS [19]. Immobilized *Aspergillus flavus* was able to decolorize and degrade Drimarene Blue dye into nontoxic metabolites [22].

Besides the use of enzymes to degrade dyes, biosorbents from the biomass of bacteria, fungi and algae have also been used to remove dyes from wastewater [23,24]. The highest decolorization efficiency was achieved by *Aspergillus fumigatus* mycelia at pH 3.0, biomass 3.0 g/L within 30 min, where – COOH ^–^ and – NH^3+^ functional groups on the mycelium surface were responsible for dye adsorption [17]. In general, dyes could be removed efficiently by *Aspergillus* using the biodegradation properties of enzymes and the biosorption capacity of mycelia [10,18,22]. We postulate that biosorption may dominate the decolorization system; however, the dye is only transferred onto the mycelium surface and remains a threat to the environment. Only nontoxic endproducts of dye degradation are considered to be acceptable and non-harmful to the environment. However, there is a paucity of data showing a correlation between the decolorization role by *Aspergillus* and the removal of dyes and a consequent improvement in wastewater quality.

In this work, *Aspergillus sp*. TS-A CGMCC 12964 (TS-A) was found to remove dye color effectively. Extracellular enzymes and mycelia from TS-A were applied separately to decolorize Mordant Yellow 1 (MY1) in the simulated dye wastewater. Biodegradation products of Mordant Yellow 1 with the extracellular enzymes were analyzed by UV-Vis spectrophotometry and FTIR analysis. Decolorization activity of TS-A was examined under different conditions, including contact period, dye concentration and pH.

## Materials and methods

### Chemicals

MY1 was purchased from Aladdin Reagent Co., Ltd. (Shanghai, China). ABTS (2, 2ʹ-azion-bis (3-ethyl-benzothiazoline)-6-sulphonic acid) and VA were purchased from Bio Basic Inc. (Canada). All of chemicals and reagents used in this study were of analytical grade.

### Microbial source and culture mediums

*Aspergillus sp*. TS-A CGMCC12964 (TS-A) was isolated from activated sludge of a textile factory in Shihezi, Xinjiang, China, and stored at the China General Microbiological Culture Collection Center (CGMCC 12964). Czapek’s medium used in this study contained 2 g/L NaNO_3_, 1 g/L K_2_HPO_4_, 0.5 g/L KCl, 0.5 g/L MgSO_4_, 0.01 g/L FeSO_4_ and 30 g/L sucrose (pH 6). All culture media were autoclaved at 120°C for 20 min before inoculation.

### Preparation of extracellular enzymes and mycelia

TS-A was inoculated in 100 mL culture medium in a 250 mL flask, and shaken at 160 rpm and 30°C for 120 h. The fermentation broth of TS-A (120 h) was centrifuged at 10,000 r/min for 10 min at 4°C, and the live mycelia were washed and stored at 4°C. The supernatant was filtered with a 0.22-µm membrane and used as the source of extracellular enzymes. Sterilized and powder mycelia were prepared from the same batch of TS-A live mycelia (120 h). Sterilized mycelia were obtained by sterilizing the collected mycelia at 120°C for 20 min. Powder mycelia were prepared from pretreated mycelia, which were dried at 80°C for 24 h, pulverized in a mortar and pestle, and then sieved into uniform particle sizes using an 80-mesh screen.

### Decolorization activity of the extracellular enzymes and mycelia

The extracellular enzymes, live mycelia, sterilized mycelia and powder mycelia were used separately to remove MY1 (50 mg/L) in 100 mL decolorization reactions. The decolorization rate of the fermentation broth of TS-A (120 h) was used as the control. All decolorization experiments of MY1 were performed at pH 6, 30°C and 160 rpm for 1 h. The degradation liquid was centrifuged at 10,000 r/min for 10 min. Supernatants were monitored by UV-Vis spectrophotometry (Spectrumlab S22PC, China). The decolorization rate was calculated using the following equation:

(1) $$Decolorization(\%)=\frac{A_{0}-A_{1}}{A_{0}}\times 100$$

where *A_0_* and *A_1_* represent the initial and final absorbance of the dye at 354 nm, respectively [25]. All the decolorization experiments were performed in triplicate and the average values were used in calculations.

### Enzymes activities and biomass assay

Activities of three lignin oxidases (MnP, LiP and Lac) were detected from 24 to 120 h during the fermentation broth of TS-A by a S22pc spectrophotometer. The determination of enzyme activities was referenced to Pan et al [25]. One unit (U) of enzyme activity was defined as the amount of enzyme required to oxidize 1 µM substrate in 1 min. In addition, the wet weight of the TS-A biomass and pH values of the fermentation broth were measured in the decolorization process of TS-A.

### Uv-Vis and FTIR spectral analysis of enzyme degradation activity

MY1 was degraded by the extracellular enzymes for 1 h in the same decolorization system. The degradation liquid was centrifuged at 10,000 r/min for 10 min. The supernatant was scanned over the range of 200–500 nm with a UV6100S UV-Vis scanning spectrophotometer (Shanghai Mapada Instruments Co., Ltd., China). The supernatant was also mixed with KBr at a ratio of 1:1000 and ground in an agate mortar. The prepared mixtures were analyzed over the wavenumber range of 4000–600 cm^–1^ by FTIR (Magna-IR 750, Thermo Nicolet) where pure KBr powder was used as the background.

### Effects on decolorization activity of extracellular enzymes and mycelia

The effect of different conditions on decolorization by the extracellular enzymes was investigated. This included characterizing the effects of Mn^2+^ (0.05, 0.1, 0.15, 0.2, 0.5, 1, 1.5 and 2.5 mM), H_2_O_2_ (0.05, 0.1, 0.15, 0.2, 0.5, 1 and 1.5 mM), time (1, 3, 5, 8, 10, 20, 30 and 60 min), dye concentrations (30, 50, 70, 90 and 110 mg/L) and pH values (3, 4, 5, 6, 7 and 8). The effects of the mycelium on decolorization were investigated without Mn^2+^ and H_2_O_2_ present.

### Kinetics and isotherm model analysis of mycelia biosorption

The Langmuir and Freundlich isotherms were used to describe the adsorption onto the surface of the mycelium. The linear formulas of the Langmuir and Freundlich isotherms are [26]:

(2) $$\frac{1}{q_{e}}=\frac{1}{q_{m}k_{L}c_{e}}+\frac{1}{q_{m}}$$

(3) $$\ln q_{e}=\frac{1}{n}\;\ln c_{e}+\ln k_{F}$$

where *C_e_* is the equilibrium dye concentration in solution (mg/L), *q_e_* is the equilibrium absorbed dye concentration on the biomass (mg/g), *K_L_* represents the Langmuir constant rate and was evaluated by the affinity of dye for mycelia (L/mg) and *q_m_* is the complete monolayer maximum capacity of the biomass (mg/g). *K_F_* and n represent the biosorption capacity and the affinity of dye for mycelia, respectively.

Different kinetic models were estimated. The linear formulas of pseudo-first-order and pseudo-second-order kinetics are expressed as [27]:

(4) $$\ln(q_{e}-q_{t})=\ln q_{e}-k_{1}t$$

(5) $$\frac{t}{q_{t}}=\frac{1}{k_{2}{q_{e}}^{2}}+\frac{t}{q_{e}}$$

where *q_e_* (mg/g) and *q_t_* (mg/g) are the amounts of mycelia adsorbed dye at equilibrium and at time *t. K_1_* (min^–1^) is the pseudo-first-order biosorption rate, *K_2_* (g/mg·min^–1^) is the equilibrium rate constant of the pseudo-second-order reaction. The biosorption effect of different MY1 concentrations on mycelia was carried out, where different isotherm models were fitted to the experiment data.

### SEM and BET analysis of mycelia

Mycelia were examined by scanning electron microscopy (SEM; JEOL, JSM-6490LV, Japan) under an acceleration voltage of 12 kV. Prior to analysis, the mycelia were dried at 383 K for 24 h and stored in a desiccator. The surface area and porous properties of mycelia were determined with BET surface area analyzer by nitrogen adsorption-desorption at 78 K (SI/MP, Quantachrome, USA).

## Results

### Dye decolorization analysis

**Figure 1.**
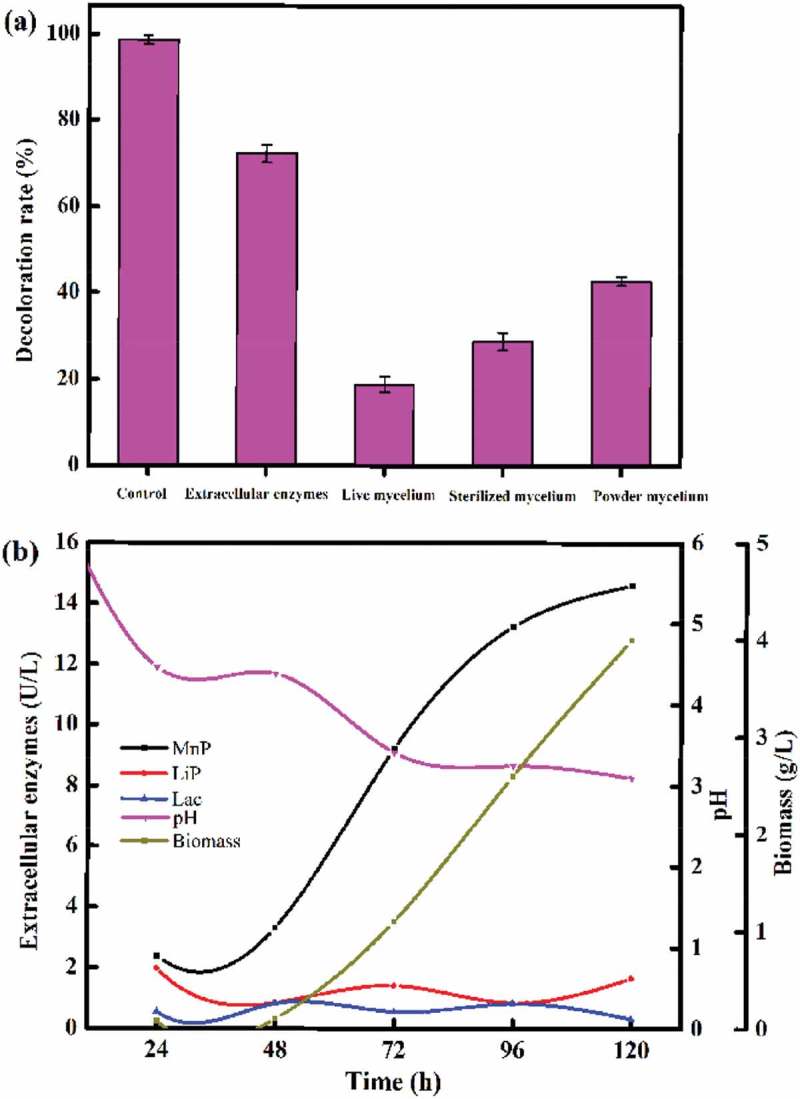
Decolorization activity and growth responses of *Aspergillus sp*. TS-A. (a) Decolorization rates of mycelia and extracellular enzymes and (b) changes of extracellular enzymes activities, biomass and pH with time. The control is the fermentation broth of TS-A.

Filamentous fungi normally display biodegradation and biosorption activities because they contain a rich source of lignin modifying enzymes and a large specific surface area. Figure 1(a) shows the biodegradation and biosorption performance of the decolorization process by TS-A. The decolorization rate reached 98.6% when MY1 was incubated with the fermentation broth of TS-A, in which over 70% was achieved by extracellular enzyme degradation of MY1 and 18.8% of MY1 was removed by live mycelia. The results showed that the decolorization rate of powder mycelia (44.2%) was higher than that of sterilized mycelia (30.6%). The powder mycelia were chosen for further adsorption experiments. Figure 1(b) describes the enzyme activities and the biomass of the TS-A fermentation broth over a period of 120 h. The activity of manganese peroxidase, which increased from 2.4 to 14.6 U/L, was more obvious than that of laccase and lignin peroxidase. The biomass of TS-A reached 4 g (wet cell)/L after 120 h. The pH of the fermentation broth decreased from 6 to 3 during the 120 h incubation period.

### Uv-Vis and FTIR analysis of the changes to the MY1 structure

**Figure 2.**
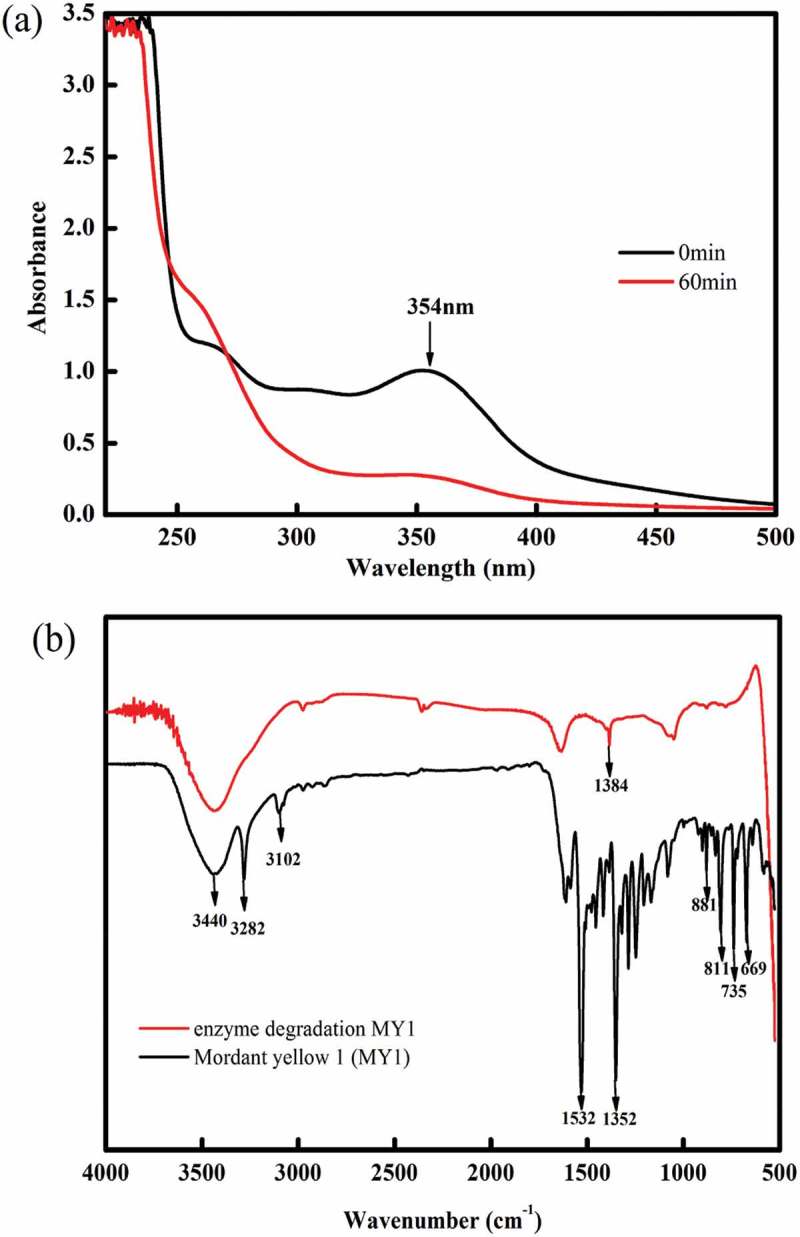
Analysis of the MY1 structure and its intermediate products. (a) UV-Vis spectrum and (b) FTIR spectra.

MY1 and its breakdown products were examined by UV-Vis and FTIR analysis (Figure 2). The UV-Vis spectra showed that the characteristic peak representing MY1 at 354 nm disappeared following incubation with TS-A (Figure 2(a)). FTIR spectra of MY1 and its breakdown products showed three groups of specific peaks over the range of 4000–600 cm^–1^ (Figure 2(b)). The first group of peaks of MY1 was observed over the broad range of 3700 to 3000 cm^–1^, which showed peaks at 3440 cm^–1^ for the asymmetric stretching vibration of – OH, and peaks at 3282 cm^–1^ and 3102 cm^–1^ that represented hydrogen stretching in the aromatic ring. The second group ranged from 2000 to 1000 cm^–1^ with peaks at 1532 cm^–1^ for N–CH_3_ bending vibrations, 1352 cm^–1^ for N = N stretching vibrations, 1352 to 1165 cm^–1^ for primary aromatic amines with C–N vibrations and 1078 cm^–1^ for the C–OH stretching vibrations. The third group of peaks ranged from 900–600 cm^–1^ with peaks at 881 cm^–1^, 811 cm^–1^ and 735 cm^–1^ for the benzene ring vibration, and at 699 cm^–1^ for C–O stretching vibrations in the benzene ring. After biodegradation with the extracellular enzymes, the peaks in the first group at 3282 cm^–1^ and 3102 cm^–1^, and in the second group at 1532 cm^–1^ and 1352 cm^–1^ were significantly reduced in intensity. The peaks in the third group at 811 cm^–1^, 735 cm^–1^ and 669 cm^−1^ were undetectable. The results showed that the azo dye MY1 could be degraded efficiently by extracellular enzymes prepared from TS-A.

### Effects of Mn^2+^ and H_2_O_2_ on MY1 degradation by the extracellular enzymes

**Figure 3.**
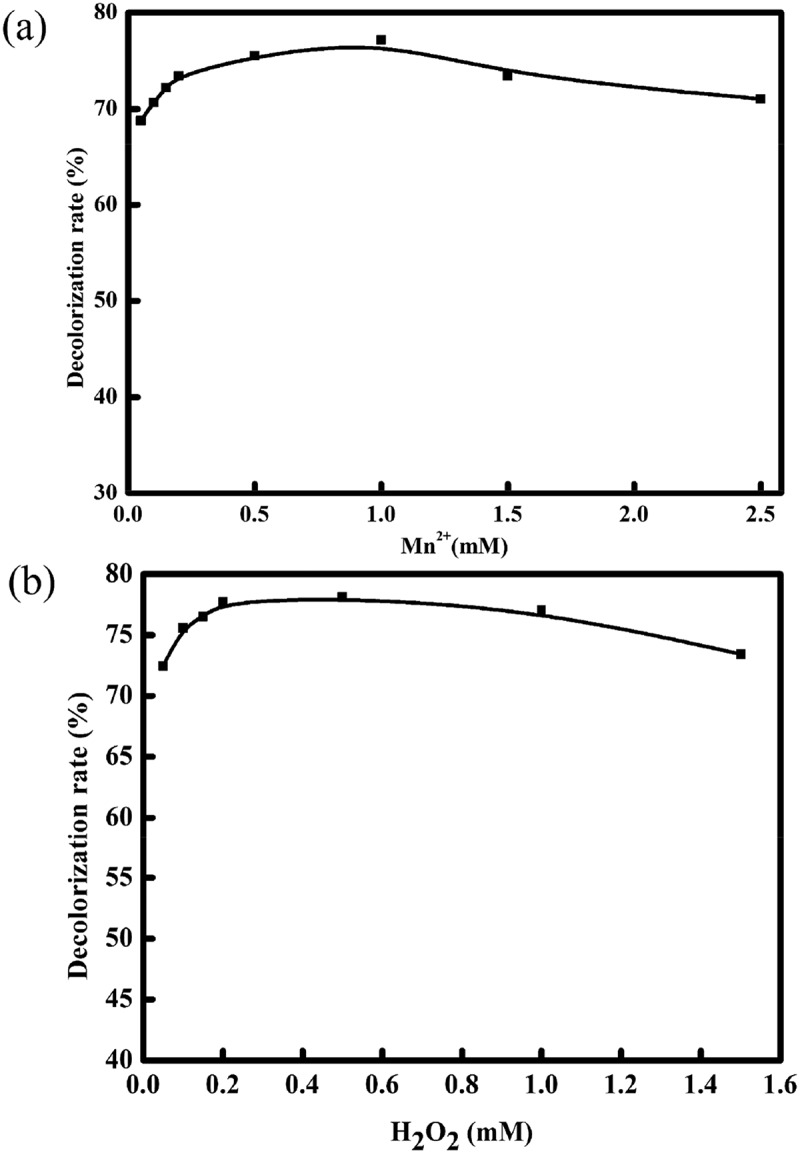
Effects of (a) Mn^2+^ and (b) H_2_O_2_ on the degradation of MY1 with extracellular enzymes.

MnPs catalyze the H_2_O_2_-dependent oxidation of Mn^2+^ to Mn^3+^, which can oxidize various pollutants [14,28,29]. LiPs were able to oxidize aromatic compounds by generating free radicals in the presence H_2_O_2_ [30]. Therefore, Mn^2+^ and H_2_O_2_ were required for the extracellular degradation system. Figure 3(a) shows that the degradation rate of extracellular enzymes increased rapidly at low concentrations of Mn^2+^ (i.e., 0.1 to 1 mM). The activity of the extracellular enzymes was inhibited when Mn^2+^ was over the optimum concentration (1 mM). The maximum degradation rate by the extracellular enzymes was 78% with increasing concentration of H_2_O_2_ (Figure 3(b)). However, the decolorization rate gradually decreased under increased concentrations of H_2_O_2_.

### Effects of time, dye concentration and pH on decolorization

**Figure 4.**
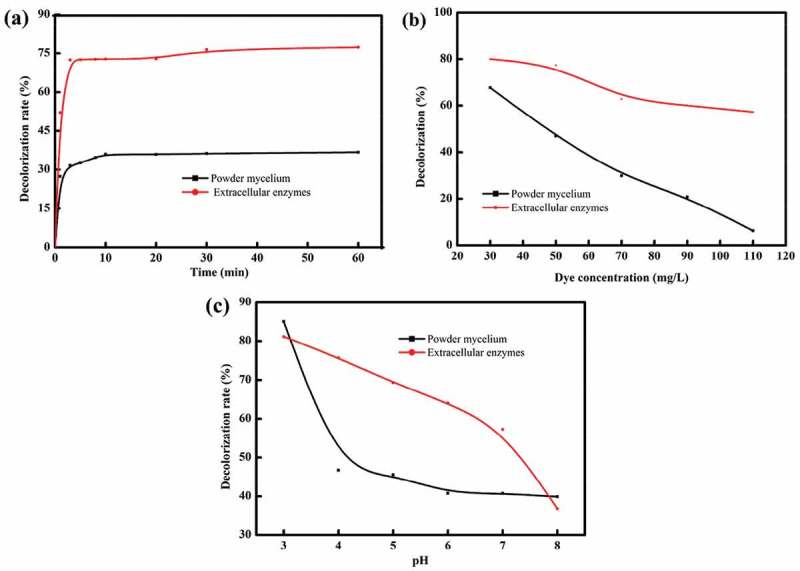
Effects of different environments on the decolorization rate of MY1. (a) Contact time, (b) dye concentration and (c) pH.

Figure 4 shows the effects of contact time, MY1 dye concentration and pH on decolorization by the powder mycelia and extracellular enzymes from TS-A. The degradation rate of MY1 by the extracellular enzymes reached 70% within 10 min and then gradually stabilized (Figure 4(a)). The decolorization rate by powder mycelia reached a relatively constant value (36.8%), indicating that the dye was no longer absorbed. The degradation rate of MY1 by extracellular enzymes decreased to 57.2% at a dye concentration of 110 mg/L (Figure 4(b)). The degradation rate of MY1 with extracellular enzymes reached 82% when the pH of the decolorization reaction was adjusted to 3 (Figure 4(c)), whereas the degradation rate decreased to 36.8% at a pH 8. This observation indicated that the extracellular enzymes showed the highest enzymatic activity under weak acidic conditions. The dye removal rate by powder mycelia was 85.1% at pH 3 and when the pH was increased above 3 the decolorization rate decreased rapidly.

### Analysis of biosorption isotherm and kinetic models

**Figure 5.**
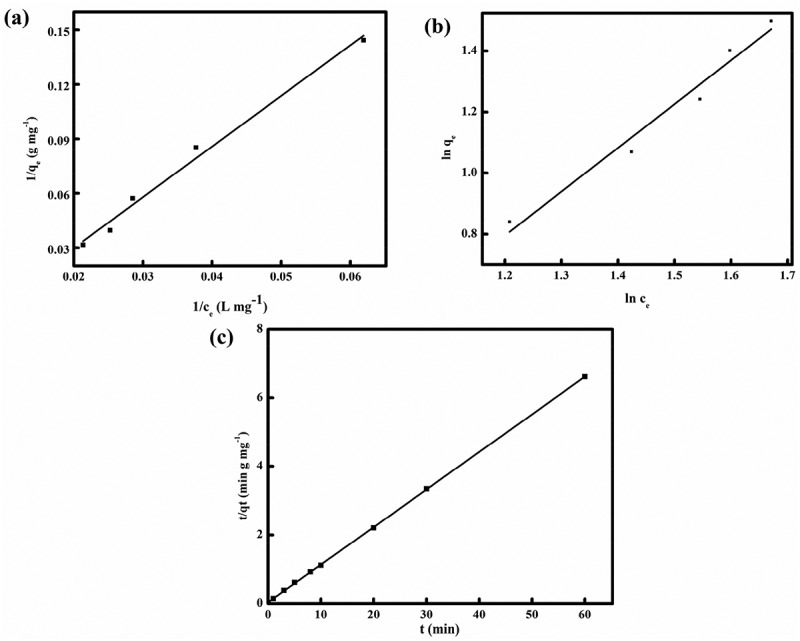
Different models for biosorption of MY1 onto pretreated mycelia: (a) Langmuir isotherm, (b) Freundlich isotherm and (c) pseudo-second-order kinetics.

In this report, two common models were used to describe the biosorption equilibrium by powder mycelia (Figure 5). The parameter *n* was > 1, which indicated a favorable biosorption process when using the Freundlich isotherms model (Table 1). Comparing the *R^2^* values between the Langmuir and Freundlich isotherms models, the Langmuir isotherm model fitted the data better than the Freundlich isotherms model to describe the biosorption behavior of powder mycelia.

**Table 1.** Isotherm constants for the biosorption of MY1 onto powder mycelia.

T (°C)	Paramater	Langmuir model	Paramater	Freundlich model
30	*q_max_*(mg·g^−1^)	38.7597	k_F_	2.5211
	k_L_	0.00925	n	1.4337
	*R*^2^	0.9867	*R*^2^	0.9631

The pseudo-second-order kinetic model was found to be suitable for describing the behavior of removing MY1 by powder mycelia (Figure 5(c)), and the data of biosorption capacity (*q_exp_*) were also close to the calculated values (*q_e,cal_*) at the set temperature (Table 2). This modeling also demonstrated that the results were well fitted (*R^2^* = 0.9999) to the pseudo-second-order kinetic model.

**Table 2.** Parameters for the two kinetic models for biosorption.

T (°C)	Kinetic models	*q_exp_* (mg·g^−1^)	*q_ecal_* (mg·g^−1^)	k	*R*^2^
30	Pseudo-first order	9.1927	476,869,998.1	0.1110	0.0915
	Pseudo-second order		9.1912	0.2372	0.9999

### SEM images and BET analysis

**Figure 6.**
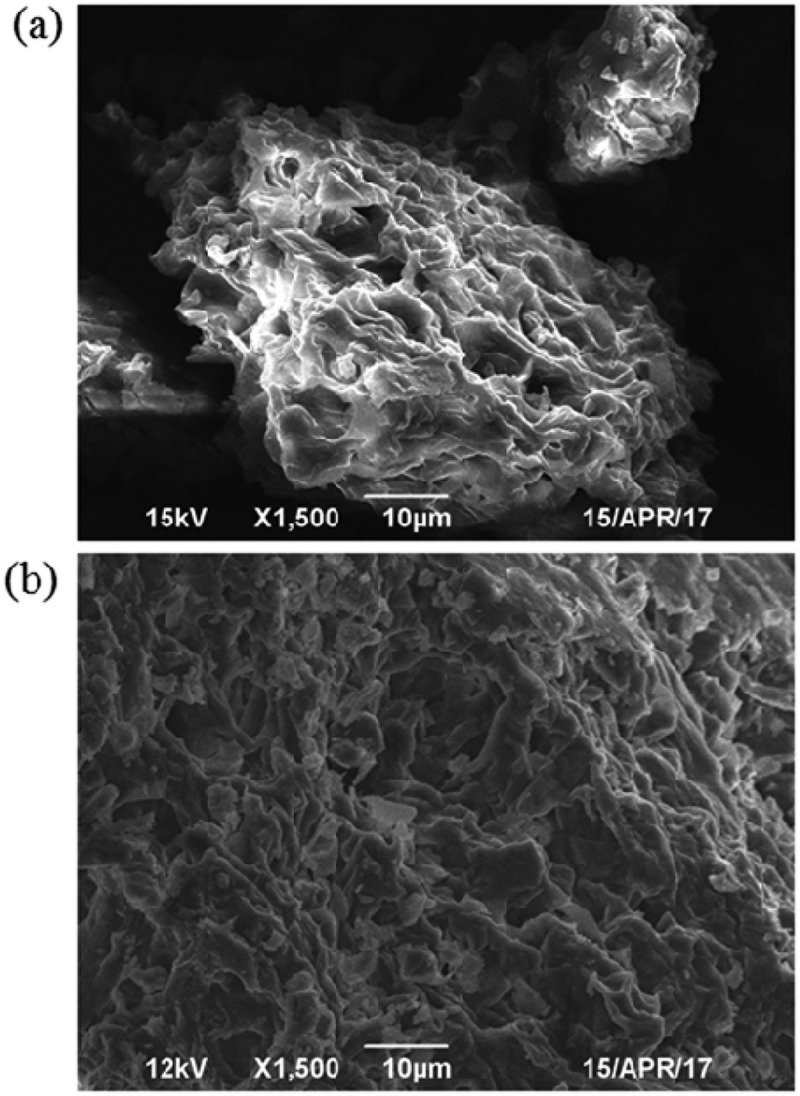
SEM images of powder mycelia. (a) Before adsorption and (b) after adsorption.

SEM images of the surface of the powder mycelia are shown in Figure 6(a). Numerous wrinkles could be observed on the surface of the mycelium, which contribute to the adsorption ability of powder mycelia. Figure 6(b) shows that many particles were found on the powder mycelium surface and these particles may represent non-dissolved dye. The BET data analysis showed that powder mycelia had a high specific surface area of 335.5 m^2^/g and an average pore diameter of 2.2 nm (Table 3).

**Table 3.** BET analyses of surface parameter for powder mycelia.

Parameters	BET surface area (m^2^/g)	Total Pore Volume (cm^3^/g)	Average Pore Size (nm)
Value	335.5	0.3	2.2

## Discussion

The major mechanism for decolorization of dye by fungi is biosorption, biodegradation or a combination of both [10]. The biosorption ability of mycelia has a strong correlation with the surface area, functional groups on the cell surface and solution pH [10,31]. Function groups such as carboxyl and amino moieties were found to be the main binding sites on the mycelium surface [32]. In this work, the decolorization rate of sterilized mycelia was higher than that of live mycelia, which showed that the intracellular enzymes contributed less to the degradation of MY1. Autoclaving of live mycelia increased the dye adsorption capacity. This observation is consistent with previous studies that showed that autoclaving ruptures fungal structures and exposes potential binding sites for dyes [32,33]. The decolorization rate of powder mycelia was higher than that of sterilized mycelia because of an increase of the mycelium surface area. The surface area and available binding sites are critical to the adsorption ability of powder mycelia. Increasing of the adsorption time did not improve the adsorption efficiency of powder mycelia because the available binding sites were saturated. Khalaf et al. reported that the adsorption ability of mycelia also depends on the equilibrium between dye molecules binding to the powder mycelium surface and those remaining in the powder mycelium pores [24]. The pH may influence the surface of the mycelium consisting of biopolymers with many functional groups [34]. Chitin, proteins, polysaccharide and other compounds on the mycelium surface act as donors of – COOH ^–^ and – NH^3+^ groups, which could bind the dye anion via electrostatic interactions [10,24]. The removal rate of MY1 with powder mycelia was 85.1% at pH 3. Similar results revealed that the biomass of *L. sajor-caju* exhibited maximum dye uptake at pH 2 [35]. Therefore, pH strongly affects the surface dye binding capacity of mycelia.

In this report, the Langmuir and Freundlich isotherms revealed that both models were applicable to describe the decolorization process. Comparison of the *R^2^* values indicated that the monolayer adsorption of MY1 played a dominant role in dye removal. Similar results were obtained in a previous report of *Aspergillus fumigatus* biosorption [17]. The pseudo-second-order kinetic model indicated that the decolorization of MY1 was determined mainly by the chemical adsorption process. The mechanism of adsorption may be described as a type of chemisorption that involves sharing or ion exchange of electrons between the surface of the powder mycelium and the dye. It was report previously that biosorption of a reactive acid dye with *Enteromorpha flexuosa* and *Gracilaria* also followed pseudo-second-order kinetics [33]. The biomass surface likely facilitates surface complexation through electrostatic attraction and ion exchange because of the presence of functional groups on the cell surface such as carboxyl, amino and phosphate moieties, and the lipid fraction [24,31].

In previous reports, the biomass from bacteria, fungi and algae had been selected as biosorbents for dye removal [7,10]. Although the biosorption capacity of biomasses can be regenerated and used for several biosorption cycles without considerable loss of biosorption ability, dye transfer onto mycelium surface is toxic and does not dissipate. Nonetheless, biosorption of toxic dyes by fungi biomasses can be removed through degradation because of the presence of active laccase, manganese peroxidase and lignin peroxidase enzymes [10,23].

In this study, biodegradation products of MY1 were monitored by UV-Vis and FTIR. Results illustrated clearly the breakdown of chromogenic groups in MY1. The change in MnP activity was more apparent than that of Lac and LiP in the fermentation broth of TS-A. A similar report revealed that laccase and manganese peroxidase were involved in the degradation process of Malachite Green by ligninolytic fungus *Aspergillus flavus* [22]. In the presence of H_2_O_2_, MnP could oxidize Mn^2+^ to Mn^3+^, which was stabilized by forming Mn^3+^ chelates as diffusing oxidizers [28,29]. LiP increased degradation capacity in the presence of H_2_O_2_ [10]. The low concentration of Mn^2+^ and H_2_O_2_ could enhance degradation of the extracellular enzyme degradation system. The activities of MnP and LiP can be inhibited when Mn^2+^ and H_2_O_2_ exceed optimum concentrations. The catalytic efficiency of MnP was dependent on the high enzyme-substrate ratio [29].

Besides substrate concentration, pH is also an important parameter that defines the decolorization activity of extracellular enzymes and powder mycelia. In this study, extracellular enzymes showed higher potential decolorization activity under weak acidic conditions. The pH-dependent activity of enzymes likely explains the importance of the pH in defining the efficiency of dye degradation. Similarly, Wen et al. reported that the degradation rate was highest when the pH was 2.96–4.8 [36]. The decrease in pH of the fermentation broth could arise from the production of acidic metabolites during the growth of TS-A. Three kinds of acidic metabolic products were examined from a *T. lixii* F21 culture extract of ARS-treated medium [12]. In general, fungi were found to grow under weak acidic conditions, which was beneficial to the degradation of dyes. In the present study, although the decolorization activity of powder mycelia exceeded that of the extracellular enzymes at pH 3, the decolorization rate of the extracellular enzymes was higher than that of powder mycelia under weak acidic conditions.

**Figure 7.**
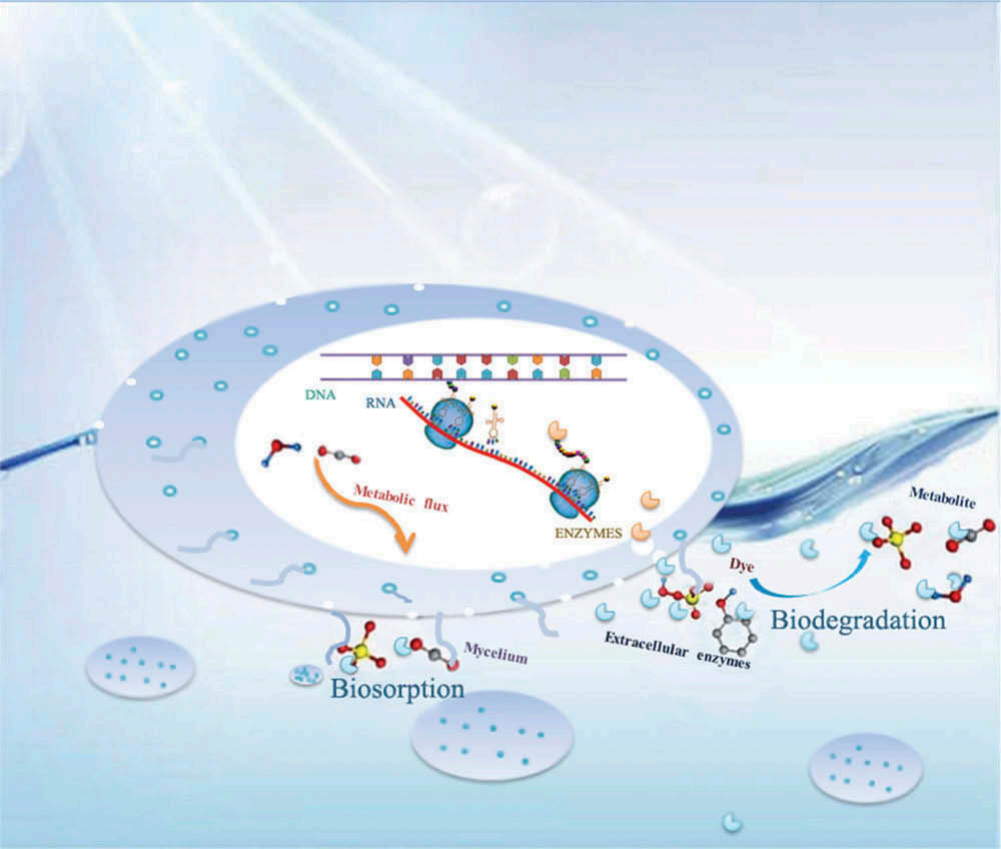
Possible decolorization process of MY1 by *Aspergillus sp*. TS-A.

As mentioned above, the decolorization process was achieved through the synergistic effects of mycelia and extracellular enzymes with the activity of the extracellular enzymes displaying the predominant role. The possible decolorization process of MY1 by TS-A is depicted in Figure 7. When the spore hypha of TS-A are inoculated in medium, active enzymes of MnP, Lac and LiP are secreted into the extracellular environment. The degradation rate of MY1 by extracellular enzymes is enhanced because of the gradual decrease in pH during the growth of TS-A. Live mycelia may behave in a particular manner that involves self-immobilization to form mycelial pellets, which have considerable mechanical strength and a large specific surface area [37]. The amino acid and polysaccharose on the surface of live mycelia provide functional groups as the main azo dye binding sites because they contain carboxyl, amino and hydroxyl groups [10,17,27]. The mycelial pellets may play a role as a bioactive carrier and gather dye molecules from dye wastewater. According to the equilibrium time of enzyme degradation and mycelium adsorption, we hypothesize that mycelia could reduce dye inhibition when extracellular enzymes degrade the high concentration of dyes during the initial incubation period. Furthermore, UV-Vis and FTIR spectra showed that dye molecules were degraded into small molecules, which might enter cells and even enter a metabolic flux until completely mineralized.

Decolorization features of TS-A could be transformed between biodegradation and biosorption under different conditions. The decolorization efficiency of *Aspergillus* could be controlled with the adjustment of pH. Extracellular enzymes performed suitable degradation activity against MY1. Thus, generation of lignin oxidase by gene recombination could be carried out in the future to investigate the specific mechanism of MY1 degradation. The activity sludge could be strengthened by adding *Aspergillus sp*. TS-A because of its excellent biosorption and biodegradation activity. *Aspergillus sp*. TS-A could also be potentially used in biofilm reactors because this species can adhere to surfaces, and thus be an effective system for degradation of azo dyes in wastewater.

## Conclusions

This work investigated the decolorization of the azo dye MY1 by extracellular enzymes and mycelia separately prepared from *Aspergillus sp*. TS-A CGMCC12964 (120 h). The synergetic effects of the extracellular enzymes and mycelia of TS-A successfully decolorized MY1 under acidic conditions. The breakdown of chromogenic groups in MY1 was examined. The biosorption behavior of TS-A was found to be a monolayer adsorption process, accompanied with particular heterogeneous adsorption. Biosorption was determined to involve primarily a chemical adsorption process. The decolorization activity of mycelia and the extracellular enzymes was clearly affected by pH. Biodegradation by the extracellular enzymes in a weak acidic environment was found to be the main decolorization process of the TS-A fermentation broth.
